# Time-Efficient Myocardial Contrast Partition Coefficient Measurement from Early Enhancement with Magnetic Resonance Imaging

**DOI:** 10.1371/journal.pone.0093124

**Published:** 2014-03-25

**Authors:** Shi-Jun Zhang, Yi-Xiang Wang, Jing Yuan, Jiyang Jin, Yuan-Cheng Wang, Di Chang, Dehe Weng, Andreas Greiser, Shenghong Ju

**Affiliations:** 1 Jiangsu Key Laboratory of Molecular and Functional Imaging, Department of Radiology, Zhongda Hospital, Medical School, Southeast University, Nanjing, China; 2 Department of Imaging and Interventional Radiology, Prince of Wales Hospital, the Chinese University of Hong Kong, Shatin, Hong Kong SAR, China; 3 Siemens Shenzhen Magnetic Resonance, Shenzhen, China; 4 Siemens AG Healthcare Sector, Erlangen, Bayern, Germany; University of Maryland, College Park, United States of America

## Abstract

**Objective:**

Our purpose was to validate an early enhancement time point for accurately measuring the myocardial contrast partition coefficient (lambda) using dynamic-equilibrium magnetic resonance imaging.

**Materials and Methods:**

The pre- and post-contrast longitudinal relaxation rates (reciprocal of T1) of the interventricular septum (R1_m_) and blood pool (R1_b_) were obtained from fifteen healthy volunteers and three diabetic patients with hypertension using two optimized T1 mapping sequences (modified Look-Locker inversion recovery) on a 3-Tesla magnetic resonance scanner. Reference lambda values were calculated as the slope of the regression line of R1_m_ versus R1_b_ at dynamic equilibrium (multi-point regression method). The simplified pre-/post-enhancement two-acquisition method (two-point method) was used to calculate lambda by relating the change in R1_m_ and R1_b_ using different protocols according to the acquisition stage of the post-enhancement data point. The agreement with the referential method was tested by calculating Pearson's correlation coefficient and the intra-class correlation coefficient.

**Results:**

The lambda values measured by the two-point method increased (from 0.479±0.041 to 0.534±0.043) over time from 6 to 45 minutes after contrast and exhibited good correlation with the reference at each time point (r≥0.875, p<0.05). The intra-class correlation coefficient on absolute agreement with the reference lambda was 0.946, 0.929 and 0.922 at the 6^th^, 7^th^ and 8^th^ minutes and dropped from 0.878 to 0.403 from the 9^th^ minute on.

**Conclusions:**

The time-efficient two-point method at 6–8 minutes after the Gd-DTPA bolus injection exhibited good agreement with the multi-point regression method and can be applied for accurate lambda measurement in normal myocardium.

## Introduction

Many previous studies have shown that the myocardial extracellular volume fraction (ECV), also known as the distribution volume fraction of contrast (V_d_) or fibrosis index, is able to provide information valuable for characterizing myocardial fibrosis and correlates well with histology findings [1]–[8]. However, understanding of the use of this index in both normal and abnormal subjects and its association with a variety of heart diseases needs to be improved. A non-invasive, reliable and time-efficient method for calculating ECV is highly desirable. The best known strategy for calculating ECV is based on estimating the myocardial partition coefficient (λ) of extracellular contrast agents as follows: ECV  =  λ · (1 - hematocrit) [3]–[5], [9], [10], where the hematocrit can be easily determined by hematological examination.

The dynamic-equilibrium cardiovascular magnetic resonance (MR) imaging method has been widely used to calculate λ and ECV [4], [7], [10]–[13] based on the longitudinal relaxation rates (R1 = 1/T1) of the myocardium (R1_m_  =  1/T1_m_) and blood (R1_b_  =  1/T1_b_) measured both before contrast injection and at plasma–tissue dynamic equilibrium of the contrast agent (the two-point method). This method is derived from a regression method requiring multiple measurements of R1_m_ and R1_b_ at dynamic equilibrium after the contrast is administered [2], [14] and is more time-efficient in the clinical setting. However, the accuracy and reliability of estimates using the two-point method compared with those using multi-point regression method have not yet been studied. In addition, ECVs calculated using the two-point method have been found to change over time [7], [12], [15]. Therefore, the most efficient and reliable timing for the post-contrast R1 measurement remains uncertain.

The Modified Look-Locker Inversion Recovery (MOLLI) has been proposed for measuring cardiac T1 during a single breath-hold [6], [16]–[18] and has been used to calculate λ in some reported studies [4], [11], [12], [15]. However, previously proposed MOLLI protocols have mostly been derived using 1.5-Tesla MR systems [19]–[21]. Although improvements have been made using 3-Tesla systems in some studies [13], [22], these protocols may still have limitations due to longer tissue T1 at 3 Tesla, including the insufficient longitudinal magnetization recovery between the inversion pulses, as well as the heart rate (HR)-dependent accuracy [13], [22].

In this study, we first optimized the MOLLI schemes for T1 mapping at 3 Tesla using a tube phantom study and then validated a two-point method MR imaging protocol for accurate and time-efficient measurement of λ from the dynamic-equilibrium at 3 Tesla. The potential mechanisms affecting time-dependent λ and the ECV estimation when using the two-point method were investigated using the normal multi-point regression method as the reference.

## Materials and Methods

### Ethics statement

The study protocol was approved by Ethical Review Board for Clinical Research of Zhongda Hospital, Southeast University, and the study was performed in accordance with the Declaration of Helsinki. All participants gave written informed consent.

### Description of MOLLI schemes

The MOLLI sampling algorithm is expressed as readouts between inversion, with the wait beats in parentheses, such as MOLLI3(3)3(3)5 [18]. The inversion times (TIs) in one MOLLI scheme were determined by the minimum inversion time (TI_minimum_), inversion time increment (TI_increment_) and individual cardiac cycle as described by Messroghli [19].

### Phantom study

To test the T1 mapping accuracy of different MOLLI schemes, nine Gd-DTPA agarose samples with T1 values of approximately 200 to 2000 msec and T2 values of approximately 170 msec (which are similar to the values reported for post-contrast blood [15]) were studied. Three groups of MOLLI approaches with gradually changing sampling algorithms (Table 1) were tested. The reference T1 values for each sample were determined using standard inversion recovery–spin echo pulse sequences (IR-SE) [23]. A “HR” produced by a simulated ECG signal was set to 70 beats per minute (bpm). The MOLLI and IR-SE sequences were repeated alternately to assess the precision of the measurements. Two MOLLI schemes were selected from this section and, along with four protocols selected from the literature (i.e., 3(3)3(3)5, 3(3)5, 5(3)3, 4(1)3(1)2 [13], [19], [21], [22]), were tested further using simulated HRs of 40 to 100 bpm.

**Table 1:** MOLLI schemes tested in the phantom study.

		Group 1	Group 2	Group 3
**TI_minimum_/TI_increment_ (msec)**		100/80	80/100/-	65/80
**MOLLI schemes**	**I-I = 12**		4(8)3	
	**I-I = 11**	3(8)3(8)5	4(7)3	4(7)3(8)1
	**I-I = 11**	3(7)3(7)5	4(6)3	4(6)3(7)1
	**I-I = 11**	3(6)3(6)5	4(5)3	4(5)3(6)1
	**I-I = 11**	3(5)3(5)5	4(4)3	4(4)3(5)1
	**I-I = 11**	3(4)3(4)5	4(3)3	4(3)3(4)1
	**I-I = 11**	3(3)3(3)5	4(2)3	4(2)3(3)1
	**I-I = 11**	3(2)3(2)5	4(1)3	4(1)3(2)1
	**I-I = 11**	3(1)3(1)5	4(0)3	4(0)3(1)1
	**I-I = 11**	3(0)3(0)5		

Note: *TI_minimum_* is the initial inversion time as realized in the first heartbeat of the first Look-Locker experiment; *TI_increment_* is the shift TI increment relative to the TI_minimum_ for acquisition in the first heartbeat of the second Look-Locker block; *I-I* represents the cardiac cycles between the adjacent inversion pulses for longitudinal relaxation.

**Figure 1:**
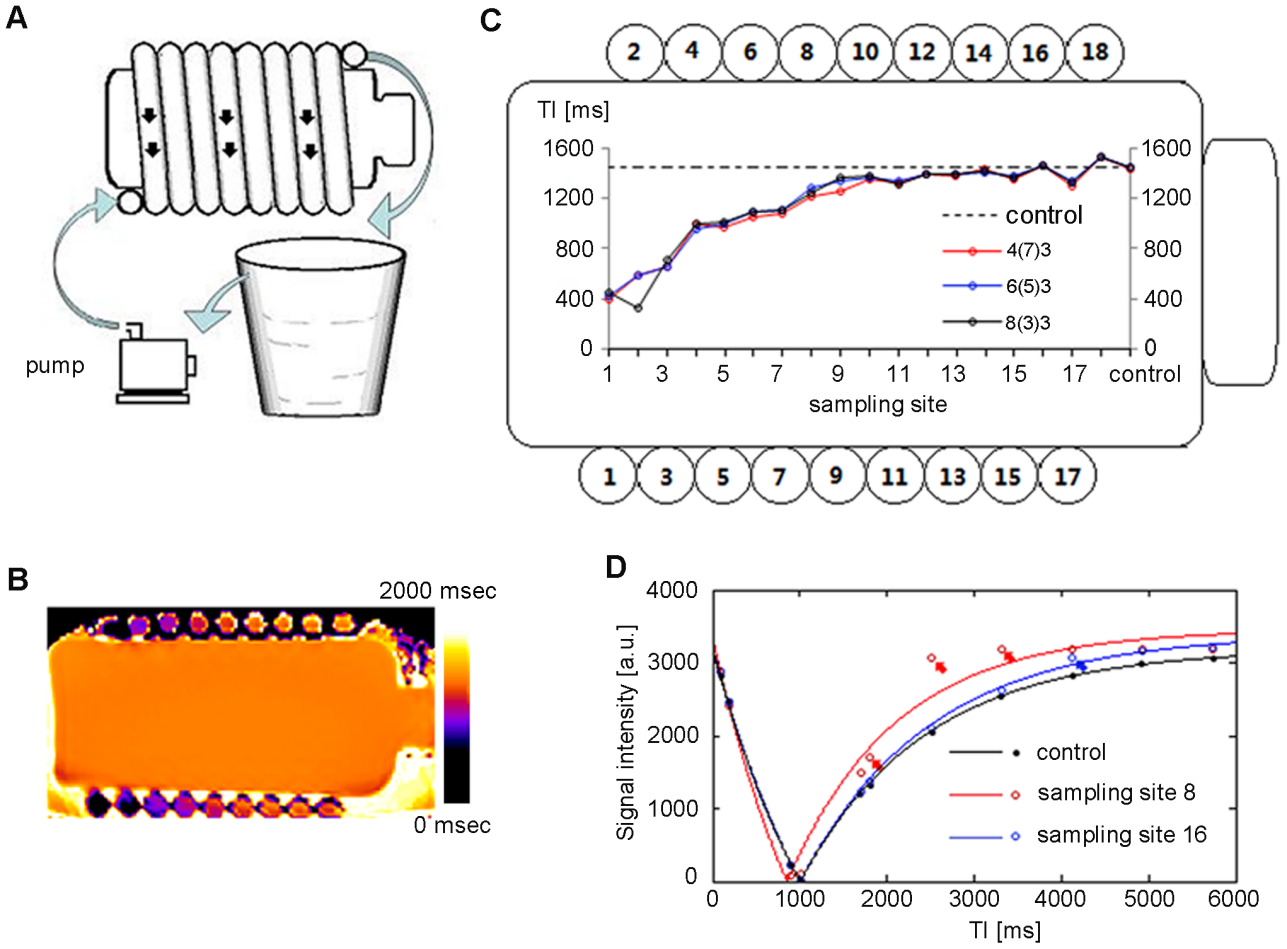
The pipeline system for testing the influence of flow on T1 mapping. A. The home-made device included the following components: a dilute bottled solution of CuSO_4_ as a control, the same solution flowing through a long silicone tube twining around the bottle, a pump providing power to the flow and a bucket outside the scanner for buffer; B. A mid-vertical T1 map of the device generated by modified Look-Locker inversion recovery (MOLLI) schemes of 4(7)3; C. MOLLI4(7)3, 6(5)3 and 8(3)3 all underestimated T1 at upstream sampling positions (sites 1–11) by a similar degree; D. The signal intensity-inversion time fitting curve deviated significantly (red arrows) at sampling site 8, whereas the skewing became very small at the downstream sampling site 16 (blue arrow).

A pipeline system phantom was designed to simulate blood flow from a peripheral region into the imaging plane during MOLLI acquisition and to study how this “flow-in effect” impacted the T1 calculation. The homemade device included a diluted CuSO_4_ solution in a bottle as the control sample, and the same solution flowed through a long silicone tube twining around the bottle, which had a T1 value that was similar to blood (Figure 1A). The flow velocity of the pipeline was set for a transit time of approximately 16 sec, which was slightly longer than the duration of the MOLLI schemes (approximately 8 cm/sec). Eighteen sampling positions along the silicone tube were simultaneously mapped within one image of the mid-vertical section (Figure 1B). Each sampling position had a different flow volume coming from out of the inversion field during MOLLI acquisition. MOLLI 4(7)3, 6(5)3 and 8(3)3 were performed in this section, with each using the same inversion intervals (11 cardiac cycles) but a different number heart beats for sampling (7, 9 and 11 beats, respectively).

### Human study

The study subjects included fifteen healthy volunteers (6 males and 9 females, aged 19 to 50 years; mean: 36.3 years) and three diabetic patients diagnosed with hypertension (1 male and 2 females, aged 56 to 69 years; mean: 62.7 years). The subjects were recruited between March and May of 2012. The diabetic patients were recruited to test the clinical feasibility of the proposed method in patients with a high risk for developing heart failure. Healthy volunteers underwent hematological examinations and twelve-lead electrocardiograms and had their histories taken prior to the study to exclude those with other diseases involving the heart. Clinical information regarding the three patients was taken from their latest medical records no more than 2 weeks before the study commenced. None of the subjects had general contraindications to cardiac MR imaging or Gd-DTPA.

**Figure 2:**
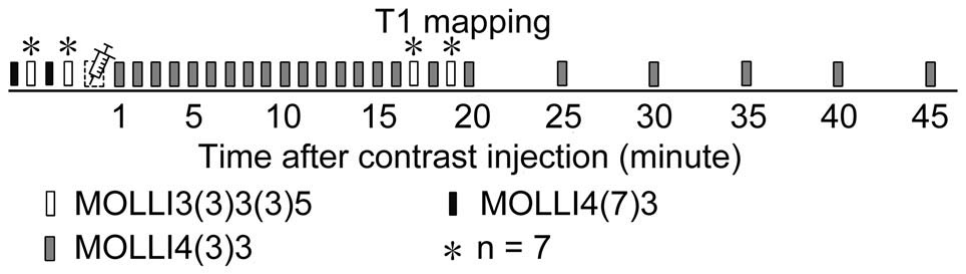
T1 mapping workflow in the human study. Measurements were repeated twice before the contrast was administered to determine T1 measurement precision, and the mean value was used to calculate λ.

All subjects received 0.15 mmol Gd-DTPA/kg body weight (Magnevist, Bayer Shering Pharma AG, Berlin, Germany), which was infused manually at a rate of approximately 0.5 ml/sec, followed by 5 ml of saline at the same rate. A mid-ventricular short axis plane was selected for imaging. T1 mapping was performed using the optimized MOLLI schemes in accordance with the imaging workflow shown in Figure 2. The commonly used MOLLI3(3)3(3)5 sequences were also performed before and after contrast injection in 7 subjects and were compared with the optimized MOLLI schemes *in vivo*. The total scan times were typically 55 to 60 minutes, including patient preparation time. Five healthy volunteers (1 male and 4 females, aged 29 to 48 years; mean: 36.8 years) participated in a repeated study 12 to 16 weeks later to assess the reproducibility of the initial results.

### MR imaging parameters

All MR scans were performed using a clinical 3.0-Tesla MR scanner (MAGNETOM Verio, Siemens AG Healthcare Sector, Erlangen, Germany). A six-channel body matrix coil and a six-channel spine matrix coil were used for signal reception. The parameters for the IR-SE for T1 measurement were as follows: repeat time/echo time = 10000/8.4 msec, number of averages  = 1, in-plane resolution  = 1.8×2.2 mm, matrix size  = 192×78 and slice thickness  = 8 mm. The TIs were 25, 50, 100, 180, 300, 500, 750, 1100, 1500, 2000, 2700, 3500, 4500, 5800 and 7500 msec. The MOLLI schemes are listed in Table 1. The following MOLLI parameters were identical between the phantom and human studies: balanced steady-state free precession readout, flip angle of 35°, parallel acquisition technique acceleration factor  = 2, 6/8 partial Fourier k-space sampling, identical spatial resolution and matrix size with IR-SE and repeat time/echo time = 2.5/1.06 msec. A three-lead wireless vector-cardiogram was used to detect the R-wave that triggered MRI acquisition. All T1 maps of the heart were gated to mid-diastole and collected under an end expiration breath hold with patients in the supine position.

### Image analyses and data processing

IR-SE T1 values were calculated by fitting signal intensities (SI) acquired at different TIs to a 2-parameter mathematical model of SI  =  SI_0_ · | 1−2 · exp (−TI/T1) |, using a home-developed MATLAB program (Version R2012a, Math Works, Natick, USA). Phantom T1 values of the two selected MOLLI schemes were fitted to the IR-SE T1 values using linear regression functions. These linear functions were used to correct the measured *in vivo* MOLLI T1 values.

In each T1 map, regions of interest (ROIs) of 2–3 square centimeters were selected from the interventricular septum and the left ventricular blood pool. The myocardium-blood border was carefully excluded from the ROIs to avoid interference from the partial volume effect. We obtained the mean T1 and R1 values within the ROIs. In each subject, the blood-tissue contrast equilibrium state was judged by two independent examiners and determined using the deviation of the R1_m_ versus R1_b_ from a linear relationship [14]. Of the two time points suggested by the two examiners, the later value was selected as the time at which individual equilibrium was established. In each subject, the linear regression of the R1_m_ and R1_b_ values, which were acquired with optimized MOLLI pre-contrast and at 6 (according to the latest equilibrium state establishment time of the 18 subjects) to 45 minutes post-contrast, was used to generate a slope as the reference λ; this approach is referred to as the ***regression method*** in this study. The ***two-point method*** was applied to calculate a series of λ values using different post-contrast data points as follows: λ  =  ΔR1_m_/ΔR1_b_, where ΔR1  =  R1_post-contrast_ − R1_pre-contrast_. The apparent λ before equilibrium establishment was also calculated using the two-point method. All calculations for λ were performed using T1 values corrected according to the functions derived from the phantom study.

### Statistical analyses

Statistical analyses were performed using SPSS statistical software (v. 18, Chicago, IL). Numerical data were reported as mean values ± standard deviation, except when otherwise specified. The coefficient of variation (CoV) for multiple repeated measurements was used to evaluate precision. For statistical comparisons, the independent-sample *t* test, paired-sample *t* test, Pearson's correlation coefficient and Bland-Altman plots were applied. The intra-class correlation coefficient (ICC) on absolute agreement was applied to assess measurement reproducibility and agreement. A *p*<0.05 was considered statistically significant.

## Results

### Phantom study

**Figure 3:**
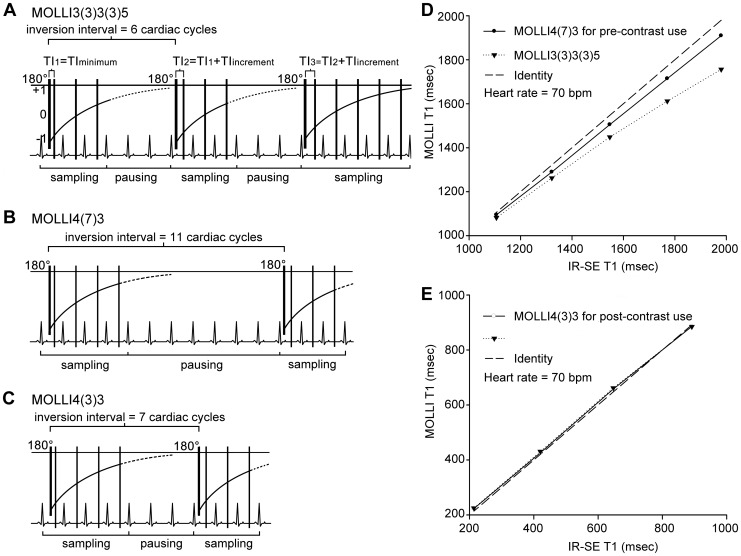
Comparison of the optimized and original modified Look-Locker inversion recovery (MOLLI) schemes. A. MOLLI3(3)3(3)5, a scheme in common use, with a duration as long as 17 cardiac cycles; B. MOLLI4(7)3 for pre-contrast use, with a shorter duration of 14 cardiac cycles and a longer inversion interval of 11 cardiac cycles; C. MOLLI4(3)3 for post-contrast use, with a shorter duration of 10 cardiac cycles and a longer inversion interval of 7 cardiac cycles; D. MOLLI4(7)3 improved the accuracy of the T1 measurement in the five samples with longer T1; E. The MOLLI4(3)3 T1 values were not significantly different from the MOLLI3(3)3(3)5 T1 values (p = 0.132, 0.115, 0.254 and 0.672 in ascending order of T1 values). Both significantly but slightly overestimated the shortest three sample T1 values and underestimated the sample T1 of 892 msec. Heart rate = 70 bpm.

**Figure 4:**
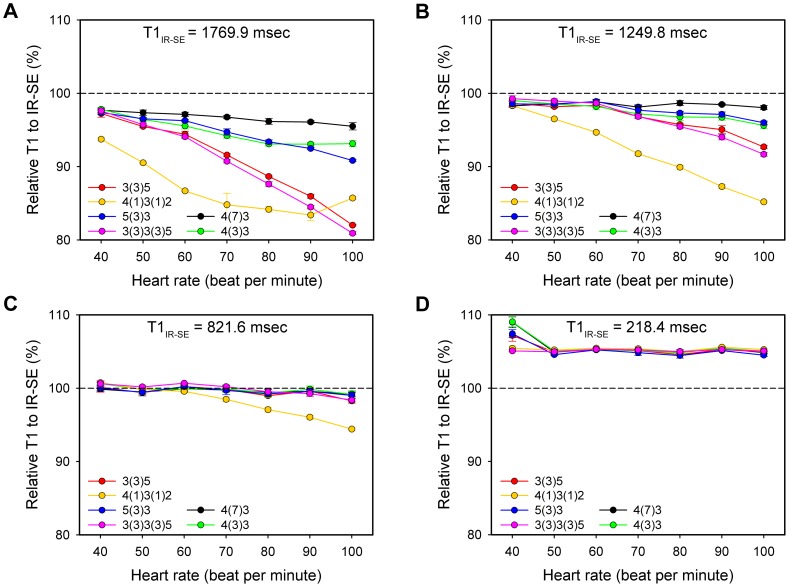
Comparison of different modified Look-Locker inversion recovery (MOLLI) sampling algorithms with inversion recovery-spin echo (IR-SE). y axis: relative T1 values (± SD) to those determined by IR-SE, as measured with different MOLLI schemes on sample T1 of 1769.9 msec (A), 1249.8 msec (B), 821.6 msec (C) and 218.4 msec (D). The measurements were repeated seven times. MOLLI4(7)3 exhibited the best heart rate (HR)-independent accuracy of the six MOLLI schemes on the two longer T1 samples, which were closed to the native blood and myocardium T1. For shorter T1, all exhibited good HR-independent accuracy except for 4(1)3(1)2.

T1 values measured using the different MOLLI schemes exhibited good agreement with the IR-SE results of the four short-T1 agarose samples (between 213 and 892 msec). Varying degrees of T1 underestimation were found in the other five samples, which all had longer T1 values (between 1107 and 1982 msec). In general, MOLLI schemes with shorter inversion intervals more profoundly underestimated T1, and the degree of underestimation (in terms of the absolute T1 value) increased as the T1 values increased (see Figure S1). After considering accuracy and breath-hold durations, the following two MOLLI schemes were selected for the *in vivo* study: 4(7)3 for the pre-contrast measurement (T1 = 1000 to 2000 msec) and 4(3)3 for the post-contrast measurement (T1 = 200 to 1000 msec) (Figure 3). These two MOLLI schemes tended to be more HR-independent than schemes reported in the literature for their respective applicable scopes (Figure 4). The following linear regression equations described the relationship between the two MOLLI sequence values and IR-SE results and were used to correct T1 values measured *in vivo*: T1_IR-SE_ = 1.07×T1_MOLLI4(7)3_ −62.4 msec (T1 = 1000∼2000 msec) and T1_IR-SE_ = 1.02×T1_MOLLI4(3)3_ −15.8 msec (T1 = 200∼1000 msec). The CoVs for repeated measurements with IR-SE, MOLLI4(7)3 and MOLLI4(3)3 (repeated 5, 8 and 8 times, respectively) were all less than 0.01, except for the MOLLI4(7)3 sequence with the smallest reference T1 (approximately 213 msec), which had a CoV of 0.0101.

In the pipeline system phantom study, the flow caused a noticeable impact on the T1 measurements, as expected (Figure 1C, 1D). Underestimation was more pronounced at upstream sampling positions (sites 1 to 8) but became negligible at downstream sampling positions (sites 9 to 18). The three MOLLI schemes used in this section exhibited similar degrees of underestimation, which suggests that increasing the number of sampled heart beats did not mitigate the effects of flow-in when calculating T1.

### Human study

#### T1 measurement with MOLLI

**Figure 5:**
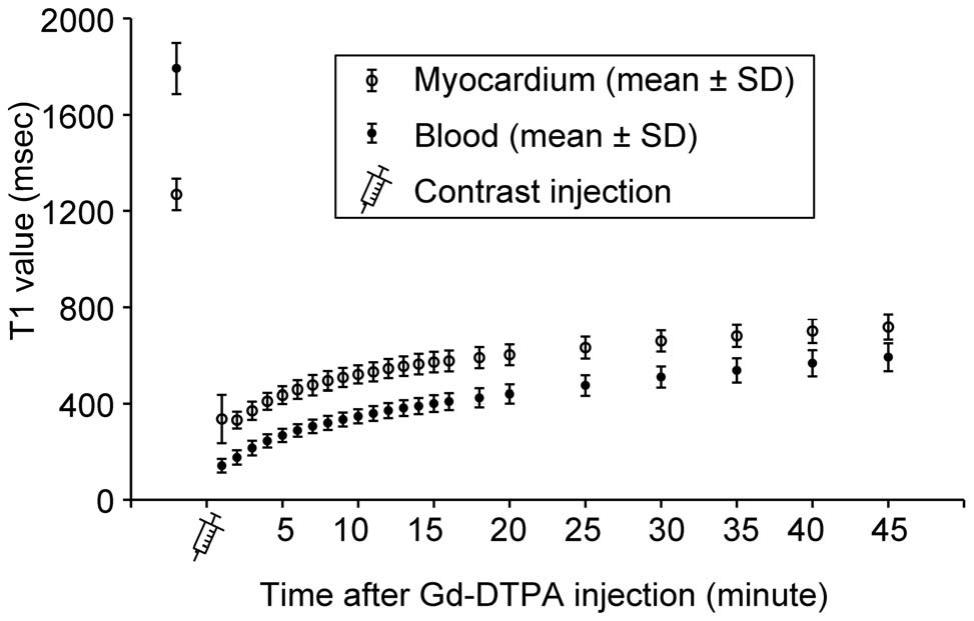
T1 of the myocardium and blood of the 18 subjects before and after Gd-DTPA injection. Rings, T1 of the myocardium; dots, T1 of blood; bars, mean ± standard deviation.

When using the optimized MOLLI method and corrected with the linear regression equations, the pre-contrast T1_m_ and T1_b_ values of all 18 participants were 1269.3±61.5 msec and 1793.4±101.4 msec, respectively. Between 1 and 45 minutes after contrast injection, the T1_m_ values rose from 336.4±96.1 msec to 718.0±47.5 msec, and the mean T1_b_ values rose from 141.5±23.8 msec to 591.9±53.7 msec (Figure 5). The *in vivo* comparisons between the optimized MOLLI schemes and MOLLI3(3)3(3)5 of the seven subjects is shown in Table 2. In results similar to the phantom study, MOLLI4(7)3 showed higher T1_m_ and T1_b_ values than MOLLI3(3)3(3)5 before contrast agent injection, whereas MOLLI4(3)3 showed smaller T1_m_ and T1_b_ values than MOLLI3(3)3(3)5 between 16 and 20 min after contrast agent injection. The breath-hold durations were reduced by 2.8±0.3 sec and 6.6±0.8 sec, for MOLLI4(7)3 and MOLLI4(3)3 respectively, compared with those of MOLLI3(3)3(3)5. The pre-contrast scan-rescan ICCs (95% confidence interval) of MOLLI4(7)3 on T1_m_ and T1_b_ were 0.956 (0.886–0.983) and 0.937 (0.840–0.976), respectively, and were comparable with those of MOLLI3(3)3(3)5, which produced ICCs of 0.989 (0.920–0.998) and 0.990 (0.949–0.998), respectively.

**Table 2:** *In vivo* comparison between optimized MOLLI and MOLLI3(3)3(3)5.

		Reference	MOLLI4(7)3	MOLLI4(3)3	MOLLI3(3)3(3)5
**Duration (sec)**		N.A.	13.2±1.6	9.4±1.2†	16.0±2.0††
**Inversion pulse interval (sec)**		N.A.	10.4±1.3	6.6±0.8†	5.7±0.7††
**Pre-contrast T1 (msec)**	**Myocardium**	1267.1±40.1	1242.5±37.5*	N.A.	1212.1±31.1*†
	**Blood pool**	1823.7±90.6	1762.7±84.7*	N.A.	1708.6±86.7*†
**Post-contrast T1 (msec)**	**Myocardium**	586.7±35.4	N.A.	590.7±34.7*	602.8±30.0*†
	**Blood pool**	426.5±25.7	N.A.	433.7±25.2*	439.5±23.7*†

Note: Data are given as the mean ± standard deviation, n = 7. Reference T1 values were measured using optimized MOLLI and corrected by the corrected function derived from the phantom study. Post-contrast T1 values were calculated as the mean values of 16, 18 and 20 min for MOLLI4(7)3 and the mean values of 17 and 19 min for MOLLI3(3)3(3)5. Significant differences in the paired sampling *t* test (*p*<0.05) are labeled by * when compared with the reference, † compared with the MOLLI4(7)3, and ‡ compared with the MOLLI4(3)3.

#### Dynamic equilibrium establishment and the regression method

**Figure 6:**
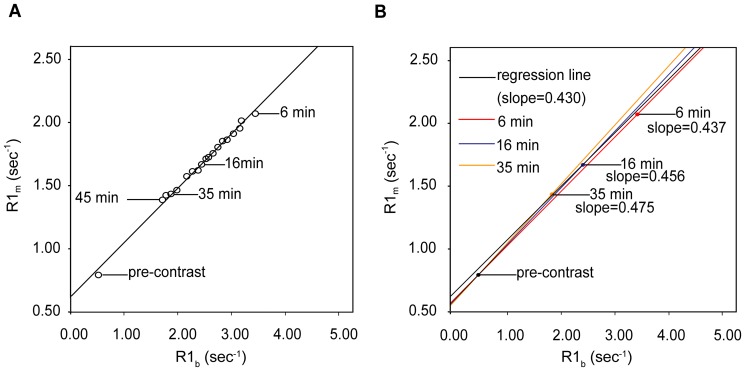
Multi-point regression and two-point methods. A. The multi-point regression method at dynamic equilibrium; B. The same data were then used to calculate λ using the two-point method with representative post-enhancement time points of 6 min, 16 min and 35 min after contrast administration. The slope of the straight line determined by the corresponding two points was used to represent the three two-point method λ values. The deviation from the regression linearity of the pre-contrast point resulted in an increase in the λ measurement over time, which was observed with the two-point method. Data were taken from a 56-year-old man with diabetes and hypertension.

The deviation from linearity of R1_m_ versus R1_b_ was significant during the first and second minutes after contrast administration and became milder from the third minute on. The deviations were negligible in 6 of the 18 cases at the fourth minute, and the deviations were negligible in 16 cases at the fifth minute. No significant deviations were found from the sixth minute on, indicating that states of dynamic equilibrium were established in all 18 cases (at 3, 2 and 5 min after contrast injection in the three patients). Interestingly, pre-contrast data points were consistently below the regression line in each subject, although the deviations were very small. An example of the regression approach is shown in Figure 6A. For the 18 participants, the r^2^ value when calculating λ using pre-contrast and 6-45 minutes post-contrast data points was 0.993±0.004 (range: 0.985–0.998), which indicated good linearity of R1_m_ versus R1_b_ values between 6 and 45 minutes following contrast; this time period was identified as the dynamic equilibrium phase.

#### Comparison of λ

**Figure 7:**
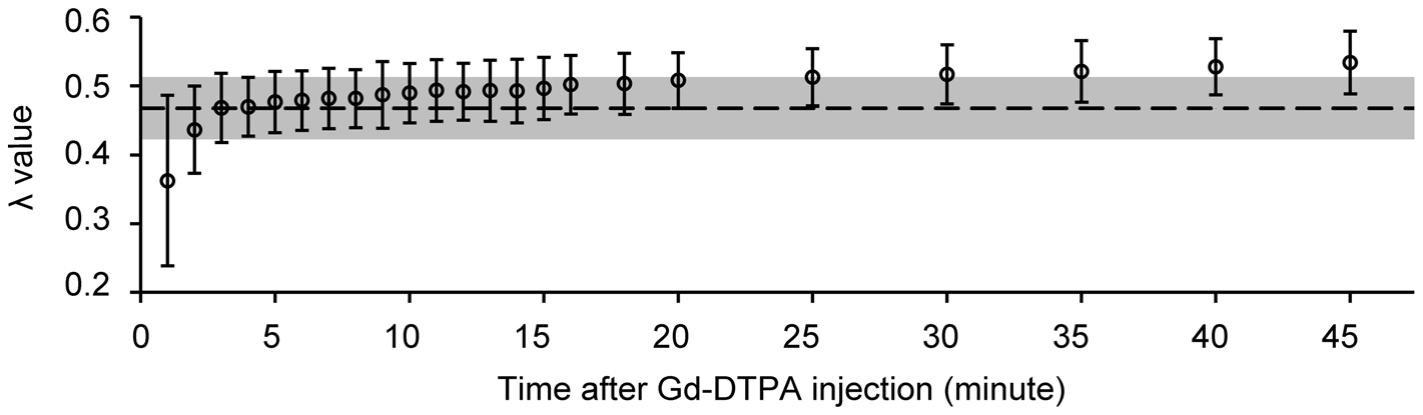
The two-point method exhibited a time-dependence in calculating the λ. The λ values were overestimated by varying degrees when using the two-point method at different stages compared with the regression method. Rings, two-point method λ; dashed line and gray box, regression method λ ± standard deviation; n = 18.

**Figure 8:**
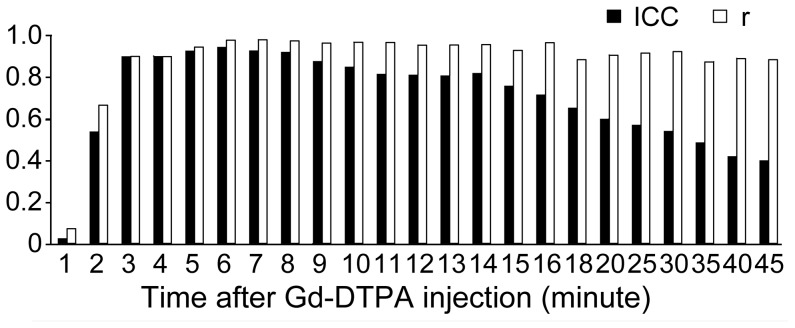
Agreement between the two-point method at different stages and the regression method. The agreement between the two methods was evaluated with an intra-class correlation coefficient (black) and Pearson's correlation coefficient (white), n = 18.

The mean regression method λ value of the 18 subjects was 0.468±0.042 (range 0.410 to 0.523), whereas the two-point method λ value increased over time following contrast, from 0.479±0.041 at the 6^th^ minute to 0.534±0.043 at the 45^th^ minute (Figure 7). An example of the two-point method using different post-enhancement data points (6 min, 16 min and 35 min) is shown in Figure 6B. Pearson's correlation coefficient (r) between the two-point method λ and the regression method λ increased with time while equilibrium was being established before the sixth minute; the coefficient remained greater than 0.875 from the sixth minute on. The ICCs on absolute agreement of the two methods were 0.946, 0.929 and 0.922 at the 6^th^, 7^th^ and 8^th^ minutes, respectively, and dropped over time from 0.878 to 0.403 from the 9^th^ minute on (Figure 8). The absolute biases (±1.96 standard deviation) for the two-point method at minutes 6 through 8 (the early stage) were 0.011±0.017, 0.014±0.016 and 0.014±0.018, respectively, as shown using Bland-Altman plots, and those values increased from 0.019±0.024 to 0.066±0.040 from the 9^th^ minute on (see Figure S2).

#### Reproducibility

In a repeat study of 5 individuals performed more than 12 weeks later, the mean interval separating the two studies was 108 days (range: 91 to 119 days). The ICC of λ on absolute agreement between the two separate studies was 0.873 using the regression method and between 0.688 and 0.943 at different stages using the two-point method (from 6–45 min). Considering a long time interval between measurements may involve changes in hematocrit, λ values were individually adjusted as follows: ECV  =  λ · (1 − hematocrit), where the hematocrit was measured 0.5–3 hours before each study. The study-restudy ICCs for ECV were then calculated, and the values for both the regression method and the two-point method were greater than 0.887 (see Figure S3).

## Discussion

In this study, we tested and verified the two-point method for calculating the partition coefficient at dynamic equilibrium after a quick intravenous bolus injection of a contrast agent. With the use of MOLLI, λ can be calculated within two single breath-hold scans before and after introduction of the contrast agent. One major finding of this study is that the results of the two-point method at the early stage of enhancement (6–8 min after contrast agent injection) exhibited excellent agreement with the results of the regression method. This finding suggests that properly decreasing post enhancement delay time not only saves scanning time but also potentially improves accuracy.

### Equilibrium establishment

Previous studies using the two-point method have not generally included measurements between minutes 5 and 10, likely to ensure full dispersion and equilibration [4], [6]–[8], [15], [24]. Lee et al. reported that dynamic equilibrium between the plasma and myocardium was reached at 8.5 min [13]. In that study, however, only three time points were established during the first 10 minutes (3.5, 5 and 8.5 min), and equilibrium may have been reached in less than 8.5 min. The present study sampled more time points during the first 10 min following Gd-DTPA injection, and the results indicated that equilibrium can be established by the sixth minute; this finding was true in all 18 cases. In the present study, we included 3 diabetic patients who had peripheral atherosclerosis and risk factors for microcirculation dysfunction in the myocardium, and dynamic equilibrium was also established within sixth minutes in these patients. Jerosch-Herold et al. reported that the apparent V_d_ agreed, within 1%, with the true ECV at times longer than 3 min following contrast bolus injection for myocardial blood flow (MBF) above 0.5 ml·min^−1^·g^−1^, using a spatially distributed model [14]. Even at an MBF of 0.25 ml·min^−1^·g^−1^, the apparent V_d_ conformed well to the true ECV after 5 min. Compared with most reports, an MBF of 0.25 ml·min^−1^·g^−1^ is extremely low for non-infarcted myocardium [25]–[29], which suggests that an early enhancement two-point method might be useful for a wider range of patients than only the normal population. In patients with severe ischemic myocardium and expected MBF lower than 0.25 ml·min^−1^·g^−1^, an additional scan at 10 to 15 min would be helpful to ensure equilibrium.

### Time dependence

Consistent with previous studies [7], [12], the results of the present study indicate that λ (or ECV) continuously increased over time when using the two-point method. This phenomenon has been observed in other studies, but the underlying reasons have not been discussed [15], [30]. Because the accuracy of MOLLI depends on measuring true T1 values and MOLLI tends to underestimate longer T1 times, the pre-contrast point is more likely to deviate from the theoretical linearity between R1_m_ and R1_b_ (the primary cause of this deviation may be the underestimation of blood T1, which will be discussed later) and leads to a time-dependent, biased estimation (Figure 6). This mechanism may explain the discrepancy among the time dependence values of λ (or ECV) reported in studies using different T1 measurement protocols [4]. However, the estimated apparent λ also increased as dynamic equilibrium was being established, but this increase cannot account for persistent time dependence after such a long delay time. Earlier post-enhancement acquisition should increase the accuracy of λ estimation when using the two-point method, as long as equilibrium has been established, as exhibited by the better agreement between the results of the two-point and multi-point regression methods in our experiments. The reason for this increased accuracy may be the greater ΔR1 at the earlier enhancement stages lowered the impact of the inaccurate precontrast T1 measurement. Alternatively, the injection of a higher dose of contrast agent is also helpful for increasing ΔR1 and therefore improves λ estimation accuracy even when post-contrast images are acquired much later than equilibrium is established. In another word, delay time and contrast dose can impact the accuracy of ECV estimates by the same way. This finding may partially explain the higher ECV estimates in the 0.1 mmol/kg group compared with the 0.15 and 0.2 mmol/kg groups in Miller's study, despite the use of a uniform and extended delay time [7].

### Optimized MOLLI schemes

Previous studies have discussed the optimization of MOLLI parameters and their reproducibility using a 1.5-Tesla MR scanner [18], [19], but those studies recommended MOLLI schemes with an inversion interval of 6 cardiac cycles. This interval may be insufficient for longitudinal magnetization recovery at 3 Tesla due to the increased T1 at higher field strengths [31]. A significant T1 underestimation was reported in a previous study by Messroghli, et al., caused by the short inversion interval [18]. Moreover, HR-dependent inversion intervals change from 4000 to 7200 msec in the HR range of 50–90 bpm when these MOLLI schemes are used, which makes HR-based T1 correction necessary [13], [18]. In contrast, much longer inversion intervals of 11 and 7 cardiac cycles (for MOLLI4(7)3 and MOLLI4(3)3, respectively) were selected in our study to allow for the full recovery of longitudinal magnetization, and as a result, the accuracy was improved. The inversion intervals are more than 7300 and 4600 msec for MOLLI4(7)3 and MOLLI4(3)3, respectively (more than 5.5 times corresponding T1_m_ and post-enhancement T1_b_ values at 3T), with HR values less than 90 bpm. At that range, the influence of HR becomes negligible; however, underestimation may still be an issue when measuring pre-enhancement T1_b_. To compensate for extended sequence duration, we reduced the number of Look-Locker inversion recovery blocks to shorten breath-hold duration. Despite the corresponding reduction in sampling points, particularly in the first heartbeat after inversion, the precision was comparable with that of the reference IR-SE.

### Half-inverted inflow effect

**Figure 9:**
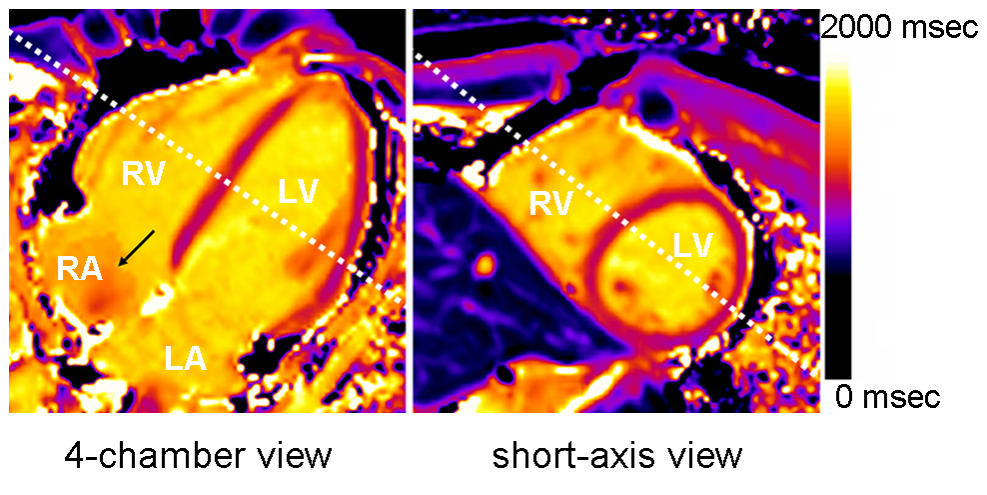
A test pre-enhancement T1 mapping with MOLLI4(7)3 using a 4-chamber view (left) and a short-axis view (right). The black arrow indicates the heterogeneity of blood T1 in the right atrium (RA). The white dashed lines indicate the relative location of each map. LV, left ventricle; LA, left atrium; RV, right ventricle.

We demonstrated, using a pipeline device, that T1 could be underestimated because of imperfect inverted blood flow to the imaged slice, similar to the inflow effect encountered when using dynamic contrast-enhanced MR imaging [32]. The underestimation of T1 depended on many factors, including flow velocity, flow direction, sequence type and repeat time. Nevertheless, we found that the T1 values measured at the downstream sampling positions (sites 12–18) were very similar to the values measured in the stable solution sample (Figure 1C), which suggests that the influence of other factors was negligible. Although a non-spatially selective inversion pulse was used, the volume excited by the inversion pulse (approximately 40–50 cm, depending on hardware) may still have been limited. During acquisition, half-inverted blood outside the inversion volume might flow into the right atrium and right ventricle (upstream positions) through the cava, producing inhomogeneous T1_b_ in the right atrium and right ventricle (Figure 9). Blood in the left atrium and left ventricle (downstream positions) came directly from the lung and right heart within 4 heart beats, which was adequate for inversion volume coverage. It is not difficult to infer from the signal recovery fitting curve of fluid (Figure 1D) that blood flow with longer T1 is more likely to be underestimated than that with a shorter T1. The significance of these results lies in the following hypotheses: (a) the blood T1 value in the heart is underestimated, (b) that underestimation may become insignificant when MOLLI is performed after contrast injection and (c) the ROI used to measure blood T1 should be set in the left ventricle, where little fresh blood may flow in during MOLLI acquisition, whereas the right ventricle is more susceptible to imperfect inverted blood flow.

### Reference method

There is still no gold standard MR protocol for calculating λ. A bolus of the extracellular contrast agent gadolinium (Gd-DTPA) followed by continuous infusion has been recommended in some previous studies as a method for achieving blood-myocardial contrast equilibrium, with a constant concentration maintained during post-contrast scanning [3], [8], [33]. This enhancement technique, however, still might bias the λ due to underestimation of the pre-contrast T1_b_. Equilibrium in our study was defined as a dynamic process, with the equilibrium concentration of the contrast dropping slowly due to renal excretion. This equilibrium allowed λ to be calculated with a wider range of R1_m_ and R1_b_ values using the regression method, which limited possible systemic errors caused by pre-contrast T1 measurement.

### Study limitations

This study has some limitations. First, there is still no gold standard method for measuring the T1 of blood. Our pipeline system demonstrated the effects of inflow on the T1 measurement, and we discussed how to reduce this influence. Second, no patient with definite scarring or severe ischemia of the myocardium was included, which limits how far our conclusions can be extended. The applicability of the two-point method at early enhancement stage within different myocardial injuries needs further confirmation.

## Conclusions

In conclusion, optimized MOLLI4(7)3 and 4(3)3 parameters are reliable for clinical use. Measurements taken using a time-efficient, two-point MRI technique at 6–8 minutes after a Gd-DTPA bolus injection exhibited good agreement with the multi-point regression method and can be applied for accurate λ and ECV measurements in normal myocardium. T1-dependent MOLLI accuracy is a key factor for the time dependence during λ and ECV measurements using the two-point method at dynamic equilibrium.

## Supporting Information

Figure S1 **Comparison of the three groups of MOLLI schemes with the IR-SE reference.** MOLLI exhibited good agreement with the IR-SE reference for shorter T1 values and underestimated longer T1 values by different degrees, which was associated with the inversion intervals. Heart rate = 70 bpm. MOLLI, modified Look-Locker inversion recovery; IR-SE, inversion recovery-spin echo.
(TIF)

Figure S2 **Bland-Altman plots of the λ measurements by the two-point and regression methods.** Each plot stands for a two-point method protocol based on a specific delay time. The solid black lines indicate the mean difference, and the dashed lines indicate the limit of agreement (mean ±1.96 standard deviation).
(TIF)

Figure S3 **Reproducibility of the λ and ECV calculated with different protocols.** Black, λ; gray, ECV; n = 5.
(TIF)

File S1 **Approval Notice from the IRB of Southeast University Zhongda Hospital.**
(PDF)

## References

[1] Kehr E, Sono M, Chugh SS, Jerosch-Herold M (2008) Gadolinium-enhanced magnetic resonance imaging for detection and quantification of fibrosis in human myocardium in vitro. Int J Cardiovasc Imaging 24: 61–68. 17429755 10.1007/s10554-007-9223-y

[2] Broberg CS, Chugh SS, Conklin C, Sahn DJ, Jerosch-Herold M (2010) Quantification of diffuse myocardial fibrosis and its association with myocardial dysfunction in congenital heart disease. Circ Cardiovasc Imaging 3: 727–734. 20855860 10.1161/CIRCIMAGING.108.842096 PMC3048790

[3] Flett AS, Hayward MP, Ashworth MT, Hansen MS, Taylor AM, et al (2010) Equilibrium contrast cardiovascular magnetic resonance for the measurement of diffuse myocardial fibrosis: preliminary validation in humans. Circulation 122: 138–144. 20585010 10.1161/CIRCULATIONAHA.109.930636

[4] Ugander M, Oki AJ, Hsu LY, Kellman P, Greiser A, et al (2012) Extracellular volume imaging by magnetic resonance imaging provides insights into overt and sub-clinical myocardial pathology. Eur Heart J 33: 1268–1278. 22279111 10.1093/eurheartj/ehr481 PMC3350985

[5] Flett AS, Sado DM, Quarta G, Mirabel M, Pellerin D, et al (2012) Diffuse myocardial fibrosis in severe aortic stenosis: an equilibrium contrast cardiovascular magnetic resonance study. Eur Heart J Cardiovasc Imaging 13: 819–826. 22634740 10.1093/ehjci/jes102

[6] Puntmann VO, Voigt T, Chen Z, Mayr M, Karim R, et al (2013) Native t1 mapping in differentiation of normal myocardium from diffuse disease in hypertrophic and dilated cardiomyopathy. JACC Cardiovasc Imaging 6: 475–484. 23498674 10.1016/j.jcmg.2012.08.019

[7] Miller CA, Naish JH, Bishop P, Coutts G, Clark D, et al (2013) Comprehensive validation of cardiovascular magnetic resonance techniques for the assessment of myocardial extracellular volume. Circ Cardiovasc Imaging 6: 373–383. 23553570 10.1161/CIRCIMAGING.112.000192

[8] White SK, Sado DM, Fontana M, Banypersad SM, Maestrini V, et al (2013) T1 mapping for myocardial extracellular volume measurement by CMR: bolus only versus primed infusion technique. JACC Cardiovasc Imaging 6: 955–962. 23582361 10.1016/j.jcmg.2013.01.011

[9] Arheden H, Saeed M, Higgins CB, Gao DW, Bremerich J, et al (1999) Measurement of the distribution volume of gadopentetate dimeglumine at echo-planar MR imaging to quantify myocardial infarction: comparison with 99mTc-DTPA autoradiography in rats. Radiology 211: 698–708. 10352594 10.1148/radiology.211.3.r99jn41698

[10] Diesbourg LD, Prato FS, Wisenberg G, Drost DJ, Marshall TP, et al (1992) Quantification of myocardial blood flow and extracellular volumes using a bolus injection of Gd-DTPA: kinetic modeling in canine ischemic disease. Magn Reson Med 23: 239–253. 1549039 10.1002/mrm.1910230205

[11] Kawel N, Nacif M, Santini F, Liu S, Bremerich J, et al (2012) Partition coefficients for gadolinium chelates in the normal myocardium: Comparison of gadopentetate dimeglumine and gadobenate dimeglumine. J Magn Reson Imaging 36: 733–737. 22488770 10.1002/jmri.23651 PMC3396792

[12] Kawel N, Nacif M, Zavodni A, Jones J, Liu S, et al (2012) T1 mapping of the myocardium: intra-individual assessment of post-contrast T1 time evolution and extracellular volume fraction at 3T for Gd-DTPA and Gd-BOPTA. J Cardiovasc Magn Reson 14: 26. 22540153 10.1186/1532-429X-14-26 PMC3405486

[13] Lee JJ, Liu S, Nacif MS, Ugander M, Han J, et al (2011) Myocardial T1 and extracellular volume fraction mapping at 3 tesla. J Cardiovasc Magn Reson 13: 75. 22123333 10.1186/1532-429X-13-75 PMC3269374

[14] Jerosch-Herold M, Sheridan DC, Kushner JD, Nauman D, Burgess D, et al (2008) Cardiac magnetic resonance imaging of myocardial contrast uptake and blood flow in patients affected with idiopathic or familial dilated cardiomyopathy. Am J Physiol Heart Circ Physiol 295: H1234–H1242. 18660445 10.1152/ajpheart.00429.2008 PMC2544489

[15] Schelbert EB, Testa SM, Meier CG, Ceyrolles WJ, Levenson JE, et al (2011) Myocardial extravascular extracellular volume fraction measurement by gadolinium cardiovascular magnetic resonance in humans: slow infusion versus bolus. J Cardiovasc Magn Reson 13: 16. 21375743 10.1186/1532-429X-13-16 PMC3059279

[16] Sparrow P, Messroghli DR, Reid S, Ridgway JP, Bainbridge G, et al (2006) Myocardial T1 mapping for detection of left ventricular myocardial fibrosis in chronic aortic regurgitation: pilot study. Am J Roentgenol 187: W630–635. 17114517 10.2214/AJR.05.1264

[17] Messroghli DR, Walters K, Plein S, Sparrow P, Friedrich MG, et al (2007) Myocardial T1 mapping: application to patients with acute and chronic myocardial infarction. Magn Reson Med 58: 34–40. 17659622 10.1002/mrm.21272

[18] Messroghli DR, Radjenovic A, Kozerke S, Higgins DM, Sivananthan MU, et al (2004) Modified Look-Locker inversion recovery (MOLLI) for high-resolution T1 mapping of the heart. Magn Reson Med 52: 141–146. 15236377 10.1002/mrm.20110

[19] Messroghli DR, Greiser A, Frohlich M, Dietz R, Schulz-Menger J (2007) Optimization and validation of a fully-integrated pulse sequence for modified look-locker inversion-recovery (MOLLI) T1 mapping of the heart. J Magn Reson Imaging 26: 1081–1086. 17896383 10.1002/jmri.21119

[20] Messroghli DR, Plein S, Higgins DM, Walters K, Jones TR, et al (2006) Human myocardium: single-breath-hold MR T1 mapping with high spatial resolution—reproducibility study. Radiology 238: 1004–1012. 16424239 10.1148/radiol.2382041903

[21] Kellman P, Arai AE, Xue H (2013) T1 and extracellular volume mapping in the heart: estimation of error maps and the influence of noise on precision. J Cardiovasc Magn Reson 15: 56. 23800276 10.1186/1532-429X-15-56 PMC3702513

[22] Kellman P, Wilson JR, Xue H, Ugander M, Arai AE (2012) Extracellular volume fraction mapping in the myocardium, part 1: evaluation of an automated method. J Cardiovasc Magn Reson 14: 63. 22963517 10.1186/1532-429X-14-63 PMC3441905

[23] Crawley AP, Henkelman RM (1988) A comparison of one-shot and recovery methods in T1 imaging. Magn Reson Med 7: 23–34. 3386519 10.1002/mrm.1910070104

[24] Nacif MS, Kawel N, Lee JJ, Chen X, Yao J, et al (2012) Interstitial myocardial fibrosis assessed as extracellular volume fraction with low-radiation-dose cardiac CT. Radiology 264: 876–883. 22771879 10.1148/radiol.12112458 PMC3426854

[25] Chareonthaitawee P, Kaufmann PA, Rimoldi O, Camici PG (2001) Heterogeneity of resting and hyperemic myocardial blood flow in healthy humans. Cardiovasc Res 50: 151–161. 11282088 10.1016/s0008-6363(01)00202-4

[26] Schindler TH, Zhang XL, Prior JO, Cadenas J, Dahlbom M, et al (2007) Assessment of intra- and interobserver reproducibility of rest and cold pressor test-stimulated myocardial blood flow with (13)N-ammonia and PET. Eur J Nucl Med Mol Imaging 34: 1178–1188. 17334762 10.1007/s00259-007-0378-5

[27] Efseaff M, Klein R, Ziadi MC, Beanlands RS, deKemp RA (2012) Short-term repeatability of resting myocardial blood flow measurements using rubidium-82 PET imaging. J Nucl Cardiol 19: 997–1006. 22826134 10.1007/s12350-012-9600-3

[28] Katoh C, Yoshinaga K, Klein R, Kasai K, Tomiyama Y, et al (2012) Quantification of regional myocardial blood flow estimation with three-dimensional dynamic rubidium-82 PET and modified spillover correction model. J Nucl Cardiol 19: 763–774. 22527800 10.1007/s12350-012-9558-1

[29] Fairbairn TA, Motwani M, Mather AN, Biglands JD, Larghat AM, et al (2014) Cardiac MR Imaging to Measure Myocardial Blood Flow Response to the Cold Pressor Test in Healthy Smokers and Nonsmokers. Radiology 270: 82–90. 24072774 10.1148/radiol.13122345

[30] Kawel N, Nacif M, Zavodni A, Jones J, Liu S, et al (2012) T1 mapping of the myocardium: intra-individual assessment of the effect of field strength, cardiac cycle and variation by myocardial region. J Cardiovasc Magn Reson 14: 27. 22548832 10.1186/1532-429X-14-27 PMC3424109

[31] Stanisz GJ, Odrobina EE, Pun J, Escaravage M, Graham SJ, et al (2005) T1, T2 relaxation and magnetization transfer in tissue at 3T. Magn Reson Med 54: 507–512. 16086319 10.1002/mrm.20605

[32] Roberts C, Little R, Watson Y, Zhao S, Buckley DL, et al (2011) The effect of blood inflow and B(1)-field inhomogeneity on measurement of the arterial input function in axial 3D spoiled gradient echo dynamic contrast-enhanced MRI. Magn Reson Med 65: 108–119. 20928889 10.1002/mrm.22593

[33] Flacke SJ, Fischer SE, Lorenz CH (2001) Measurement of the gadopentetate dimeglumine partition coefficient in human myocardium in vivo: normal distribution and elevation in acute and chronic infarction. Radiology 218: 703–710. 11230643 10.1148/radiology.218.3.r01fe18703

