# Correction: Time-Efficient Myocardial Contrast Partition Coefficient Measurement from Early Enhancement with Magnetic Resonance Imaging

**DOI:** 10.1371/journal.pone.0105133

**Published:** 2014-08-05

**Authors:** The PLOS ONE Staff

There are errors in Table 1. The authors have provided a corrected version of Table 1, which can be viewed here.

**Table 1:** MOLLI schemes tested in the phantom study.

		Group 1	Group 2	Group 3
**TI_minimum_/TI_increment_ (msec)**		100/80	80/100	65/80
**MOLLI schemes**	**I-I = 12**		4(8)3	
	**I-I = 11**	3(8)3(8)5	4(7)3	4(7)3(8)1
	**I-I = 10**	3(7)3(7)5	4(6)3	4(6)3(7)1
	**I-I = 9**	3(6)3(6)5	4(5)3	4(5)3(6)1
	**I-I = 8**	3(5)3(5)5	4(4)3	4(4)3(5)1
	**I-I = 7**	3(4)3(4)5	4(3)3	4(3)3(4)1
	**I-I = 6**	3(3)3(3)5	4(2)3	4(2)3(3)1
	**I-I = 5**	3(2)3(2)5	4(1)3	4(1)3(2)1
	**I-I = 4**	3(1)3(1)5	4(0)3	4(0)3(1)1
	**I-I = 3**	3(0)3(0)5		

Note: *TI_minimum_* is the initial inversion time as realized in the first heartbeat of the first Look-Locker experiment; *TI_increment_* is the shift TI increment relative to the TI_minimum_ for acquisition in the first heartbeat of the second Look-Locker block; *I-I* represents the cardiac cycles between the adjacent inversion pulses for longitudinal relaxation.
