# Supplementary figures and images for: Time-Efficient Myocardial Contrast Partition Coefficient Measurement from Early Enhancement with Magnetic Resonance Imaging
Source: PLoS One. 2014 Mar 25;9(3):e93124. doi: 10.1371/journal.pone.0093124 (PMC3965516; doi:10.1371/journal.pone.0093124)

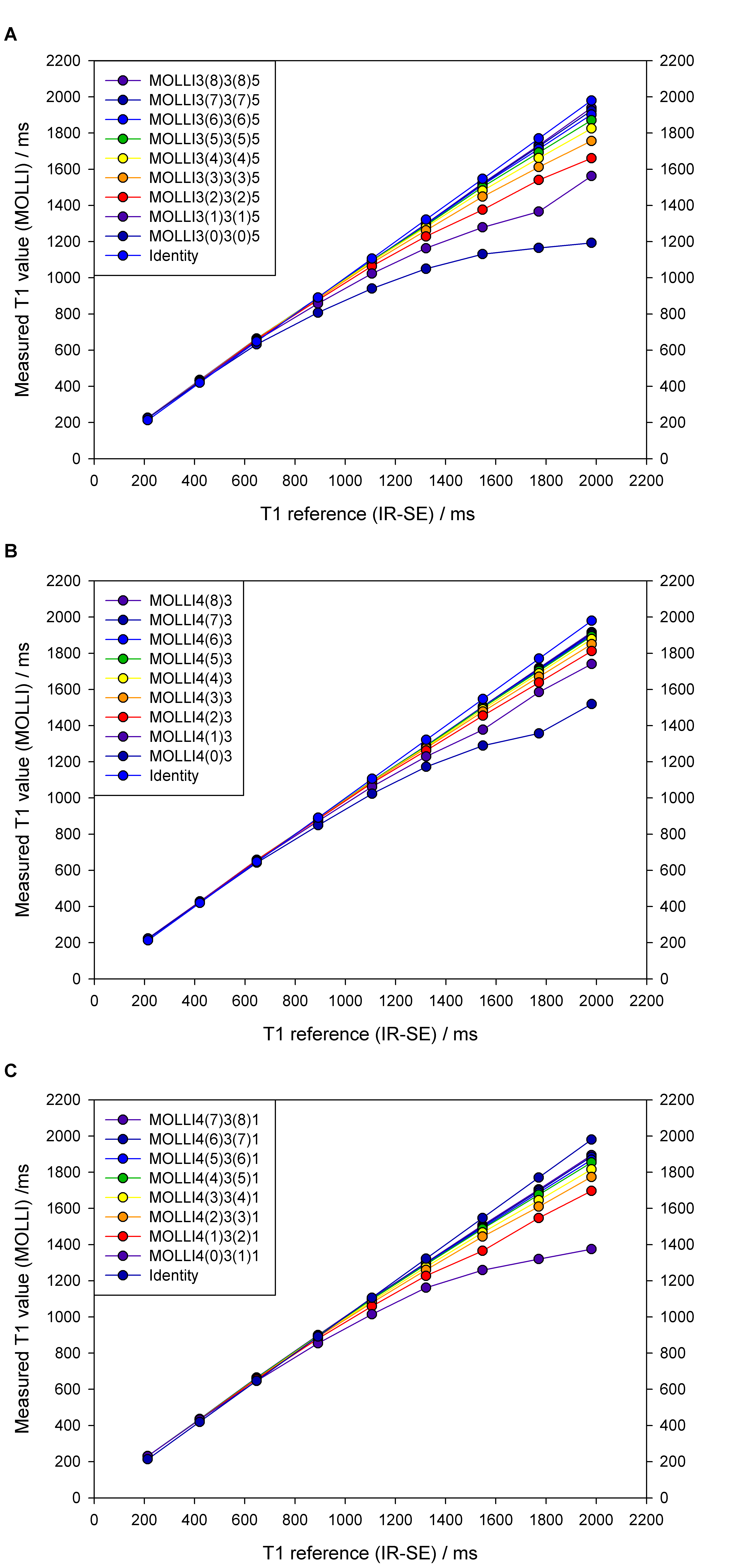

Supplement: Figure S1 — Comparison of the three groups of MOLLI schemes with the IR-SE reference. MOLLI exhibited good agreement with the IR-SE reference for shorter T1 values and underestimated longer T1 values by different degrees, which was associated with the inversion intervals. Heart rate = 70 bpm. MOLLI, modified Look-Locker inversion recovery; IR-SE, inversion recovery-spin echo. (TIF) [file pone.0093124.s001.tif]

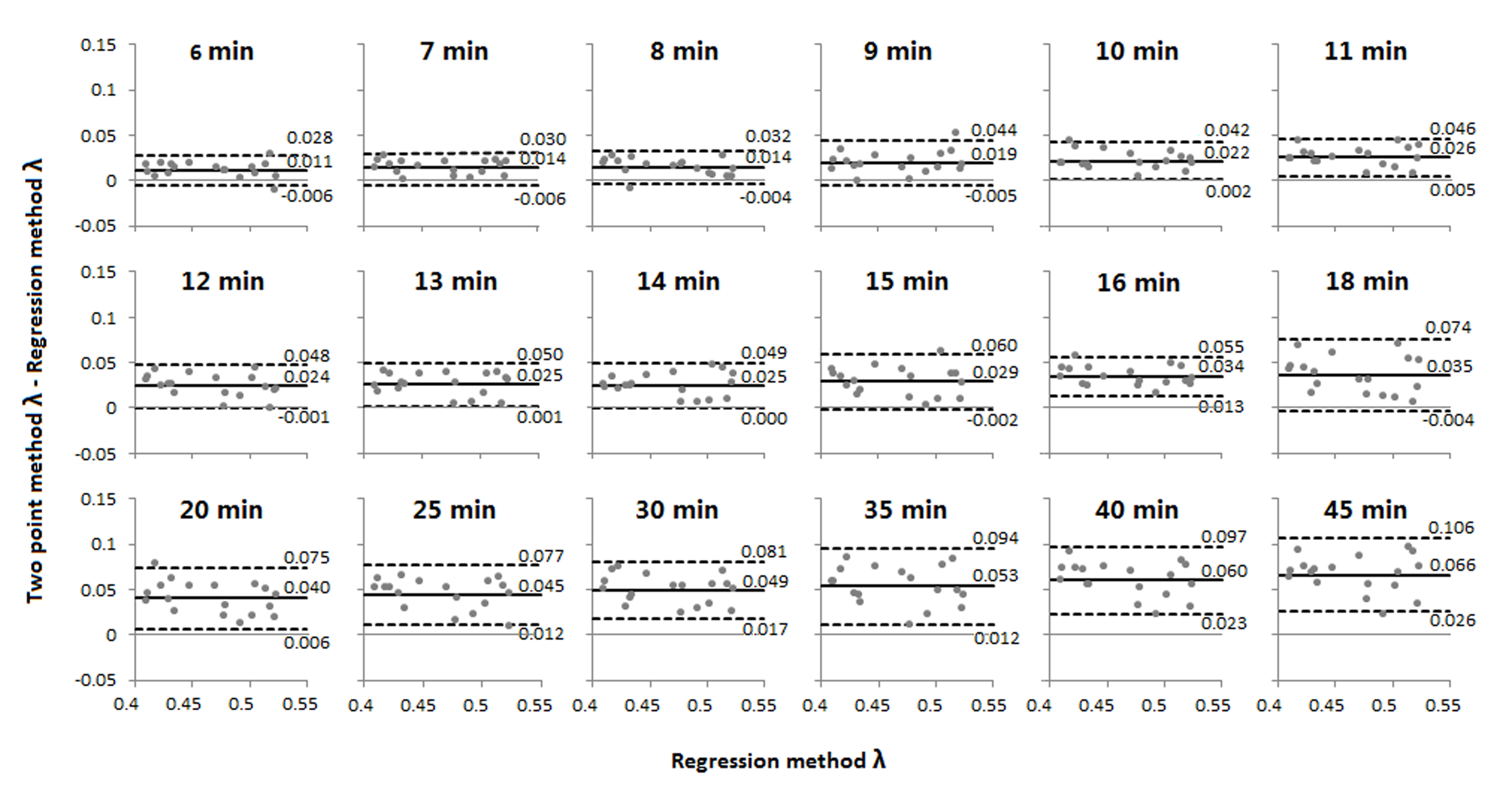

Supplement: Figure S2 — Bland-Altman plots of the λ measurements by the two-point and regression methods. Each plot stands for a two-point method protocol based on a specific delay time. The solid black lines indicate the mean difference, and the dashed lines indicate the limit of agreement (mean ±1.96 standard deviation). (TIF) [file pone.0093124.s002.tif]

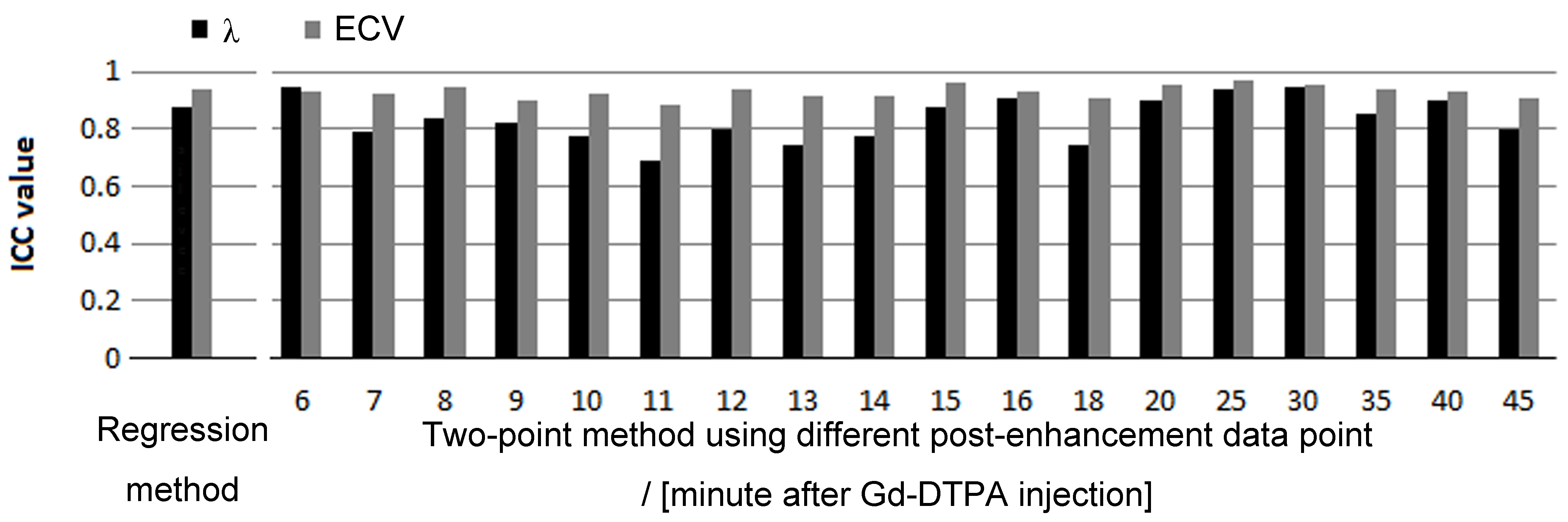

Supplement: Figure S3 — Reproducibility of the λ and ECV calculated with different protocols. Black, λ; gray, ECV; n = 5. (TIF) [file pone.0093124.s003.tif]
