# Supplementary material for: Time-Efficient Myocardial Contrast Partition Coefficient Measurement from Early Enhancement with Magnetic Resonance Imaging
Source: PLoS One. 2014 Mar 25;9(3):e93124. doi: 10.1371/journal.pone.0093124 (PMC3965516; doi:10.1371/journal.pone.0093124)
Supplement: File S1 — Approval Notice from the IRB of Southeast University Zhongda Hospital. (PDF) [file pone.0093124.s004.pdf]

东南大学附属中大医院临床研究伦理委员会
伦理审查批件
Approval Notice of IRB of Southeast University Zhongda Hospital

批件号 Approval No：2012ZDllKY01.0　　**审查日期** Date of Review：2012 年 3 月 1 日

**项目名称** Study Title	磁共振对正常人心脏功能和心肌状态相关指标的调查研究
**研究性质** Type of Research：<br>涉及人类受试者的临床研究	**伦理审查方式** Mode of Review：<br>初次审查
**主要研究者** Principal Investigator：<br>居胜红　教授	**申办方** Sponsor：<br>东南大学附属中大医院

审查文件 Documents for Review：

1、试验方案
2、知情同意书
3、招募广告
4、主要研究者履历

会议审查到会医学伦理委员会委员 Members of IRB Attending Meeting Review：

陈宝安（主席），许德义(药理，外单位)，改宏斌（律师，外单位）， 刘锦华(社区代表，外单位)，唐洪丽，景亮，杨莉，王美美，王慧萍

投票结果 the Result of Voting：

医学伦理委员会对送审文件进行审阅和讨论，参加会议人数 9 人，其中投票人数 9 人，回避人数 0 人。 投票情况：同意 0 票，作必要修改后同意 9 票，作必要修改后再讨论 0 票，不同意 0 票，终止或暂停已批准的试验 0 票。 **投票结果：作必要修改后同意**

评审意见 Comments：

同意东南大学附属中大医院放射科开展"磁共振对正常人心脏功能和心肌状态相关指标的调查研究"。并对知情同意书作适修改。

本研究结束后，请主要研究者向伦理委员会提交一份研究总结报告。

主席签名 Signature Chair：

签名日期 Date：2013-3-5

东南大学附属中大医院临床研究伦理委员会（盖章）

地址:南京鼓楼区丁家桥 87 号，邮码：210009　　**联系电话：025-83272064**

申明： 本伦理委员会的职责、人员组成、操作程序及记录遵循 ICH-GCP/中国 GCP、中国的相关法律和法规。

尊敬的东南大学附属中大医院临床研究伦理委员会：

现就伦理委员会会议结果与伦理审查批件（批件号：2012ZDllKY01.0）的要求对知情同意书进行修改，具体内容如下：

1、增加对比剂用量不高于心脏大血管磁其振增强扫描推荐用量的说明。
2、对检查时间长度进行具体说明。
3、对正常人关于补贴办法修改为“提供交通、工时等补贴 500 元”，对中途退出者要说清补贴方法。
4、写明项目由中大临床研究伦理委员会批准。

特此说明。

附件：修改后的知情同意书

东南大学附属中大医院
放射科 [illegible]

2012 年 3 月 2 日

回　执

☑ **同意**　☐ **不同意**　东南大学附属中大医院放射科开展“磁共振对正常人心脏功能和心肌状态相关指标的调查研究”。

主席签名：[illegible]

日期：2012 年 3 月 2 日

东南大学附属中大医院临床研究伦理委员会（盖章）
